# Research advances in the role of endogenous neurogenesis on neonatal hypoxic-ischemic brain damage

**DOI:** 10.3389/fped.2022.986452

**Published:** 2022-10-10

**Authors:** Andi Chen, Xiaohui Chen, Jianhui Deng, Xiaochun Zheng

**Affiliations:** ^1^ Department of Anesthesiology, Shengli Clinical Medical College of Fujian Medical University, Fujian Provincial Hospital, Fuzhou, China; ^2^ Fujian Emergency Medical Center, Fujian Provincial Key Laboratory of Emergency Medicine, Fujian Provincial Key Laboratory of Critical Care Medicine, Fujian Provincial Co-Constructed Laboratory of “Belt and Road”, Fuzhou, China

**Keywords:** neonates, hypoxic-ischemic brain damage, neurogenesis, neural stem cell, subventricular zone, subgranular zone

## Abstract

Hypoxic-ischemic brain damage (HIBD) is the main cause of perinatal mortality and neurologic complications in neonates, but it remains difficult to cure due to scarce treatments and complex molecular mechanisms remaining incompletely explained. Recent, mounting evidence shows that endogenous neurogenesis can improve neonatal neurological dysfunction post-HIBD. However, the capacity for spontaneous endogenous neurogenesis is limited and insufficient for replacing neurons lost to brain damage. Therefore, it is of great clinical value and social significance to seek therapeutic techniques that promote endogenous neurogenesis, to reduce neonatal neurological dysfunction from HIBD. This review summarizes the known neuroprotective effects of, and treatments targeting, endogenous neurogenesis following neonatal HIBD, to provide available targets and directions and a theoretical basis for the treatment of neonatal neurological dysfunction from HIBD.

## Introduction

Neonatal hypoxic-ischemic brain damage (HIBD), which is caused by perinatal asphyxia, is a primary etiology for acute neonatal mortality and long-term infant neurological dysfunction (1–3). The incidence of neonatal HIBD is 1–3 per 1,000 in developed countries, while in developing countries the rate can be as high as 25 per 1,000 (4, 5). HIBD can lead to irreversible neurological injuries including cerebral palsy, audiovisual impairment, memory difficulties, and cognitive dysfunction (6, 7). At present, standard global treatments for neonatal HIBD are focused on alleviating symptoms (e.g., hyperbaric oxygen therapy, therapeutic hypothermia, rehabilitation training); thus, there remains marked room for improved treatments (8–10).

**Figure 1:**
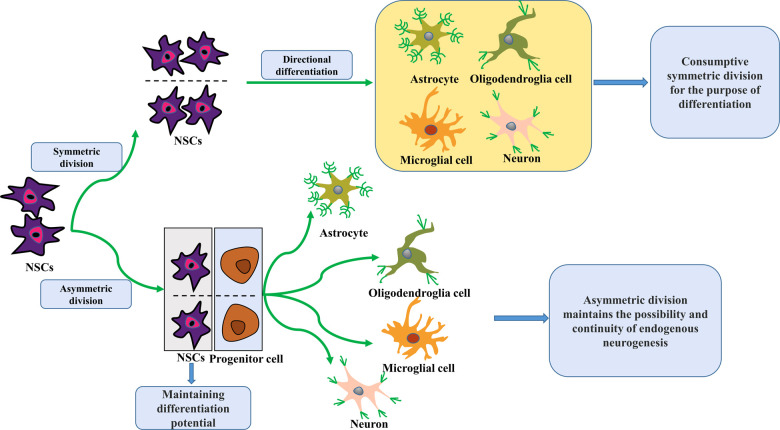
Schematic of NSCs division and differentiation. NSCs proliferate and differentiate by symmetrical and asymmetrical division. In the adult brain, they usually undergo symmetric divisions to promote self-proliferation or directed differentiation. Over time, this increasing number of expendable symmetric divisions for differentiation purposes will result in a gradual NSCs reduction, leading to a lack of spontaneous endogenous neurogenic repair capacity after brain injury. In contrast, asymmetric division maintains the possibility and continuity of endogenous neurogenesis because it retains NSCs with differentiation potential. NSCs, neural stem cells.

Endogenous neurogenesis is the process by which neural stem cells (NSCs) undergo symmetric and asymmetric divisions, after which they proliferate and subsequently differentiate into directed progenitor cells that gradually migrate to functional brain areas while undergoing uninterrupted plastic changes and establishing synaptic connections to produce neurological functions (11) (Figure 1). Under normal conditions, NSCs at neurogenesis sites remain in a resting state. When the brain is damaged by hypoxia and ischemia, they can be stimulated to proliferate, migrate, differentiate and integrate, activating brain neurogenesis. While neonatal HIBD has been shown to activate endogenous brain neurogenesis, spontaneous endogenous brain neurogenesis is limited and insufficient to fully compensate for neurons lost to injury (12). Thus, therapeutic techniques to promote endogenous neurogenesis would be of great clinical value for ameliorating neurological dysfunction from neonatal HIBD. This review summarizes the known neuroprotective effects and treatments targeting endogenous neurogenesis in neonatal HIBD, to provide available targets and directions and a theoretical basis for the treatment of neurological dysfunction from neonatal HIBD.

## Overview of endogenous neurogenesis

Over recent years, our understanding of spontaneous endogenous neurogenesis shifted from its existence exclusively during embryonic and prenatal mammalian development (13) to be present within the adult mammalian brain, NSCs concentrated primarily in the subgranular zone (SGZ) of the hippocampal dentate gyrus and the subventricular zone (SVZ) of the lateral ventricle (14). NSCs are a class of primitive “mother” cells that can give rise to neurons and glial cells, with the potential for multidirectional differentiation and the ability to maintain self-renewal (15). The NSCs population is represented by radial glial-like cells that produce proliferating intermediate progenitor cells with transient expansion characteristics, which then differentiate into neuronal cells and finally develop into mature dentate granule neurons that integrate into neural circuits of the brain to perform neurological functions (16). Due to the asymmetric division of NSCs, two cellular pools exist in the progeny of a single clone of NSCs: mature neurons and undifferentiated neural progenitor cells. The latter retains the potential for multidirectional differentiation (17). Thus, asymmetric NSCs division allows the possibility of endogenous neurogenesis.

NSCs differentiation depends mainly on the components of their microenvironment, including neuronal cells, stromal cells, and the extracellular matrix. Based on these factors, NSCs of the same origin can differentiate into different cell types, including astrocytes, oligodendrocytes, and neurons (18). Next, the migration of progenitor cells differentiated from NSCs also depends on their brain location. In the SGZ, they migrate to the molecular layer of the hippocampal dentate gyrus and eventually integrate into the neural circuits of the hippocampus, playing an important role in learning and memory (19, 20). In the SVZ, they form migratory streams in the surrounding astroglial precursor structures, and subsequently, migrate to the olfactory bulb and differentiate into intermediate neurons, ultimately participating in olfaction processes and influencing plasticity of olfactory-related behaviors (14, 21). After brain injury, progenitor cells from the neurogenesis sites migrate to damaged areas (e.g., striatum, cerebral cortex, hypothalamus) to repair damaged neurological functions by transforming into new neurons within these areas (22). This self-healing brain mechanism is enormously significant for the recovery of neurological functions after brain injury.

Although endogenous neurogenesis can improve neurological dysfunction by replacing neurons lost to injury, this is not always the case. As endogenous neurogenesis is a highly regulated process, exposure to a hypoxic-ischemic environment may lead to abnormalities in its course. In some cases, neurodevelopmental dysplasia may be a secondary process leading to brain dysfunction (23), like dysfunction of endogenous neurogenesis in hippocampal SGZ is a major factor in the development of dementia after a stroke in older adults. Current studies on the side effects of endogenous neurogenesis have focused on brain exposure to different pathological conditions in which new hippocampal SGZ granule neurons may develop abnormal morphology, leading to changes in hippocampal structure and thus ongoing exacerbation of neurological damage (24). Therefore, considering both the advantageous and deleterious effects of endogenous neurogenesis, clinical treatment of neonatal HIBD should promote endogenous neurogenesis to improve the intracranial microenvironment to facilitate the repair of damaged neurological function.

## Endogenous neurogenesis after neonatal HIBD

It was recently shown that post-neonatal HIBD can induce NSCs proliferation in neurogenesis sites, with subsequent migration of proliferating progenitor cells to a damaged brain region where they acquire the desired phenotype; furthermore, in the damaged brain region, new neurons differentiated from NSCs can integrate into functional neural loops and repair damaged nerves (25). Plane et al. (26) used 5-Bromodeoxyuridine (BrdU) as a marker of proliferating cells in the Rice-Vannucci neonatal rat model of brain hypoxia-ischemia, in which they demonstrated a significant increase in BrdU-positive cells in damaged brain areas, mainly the SVZ. Others (27, 28), using a perinatal model of severe asphyxia to investigate neurogenesis in the neonatal rat brain, noted that 3H-deoxythymidine (3H-T)-labeled brain cells revealed specific upregulation of proliferation, but only in the injured hippocampus at five days post-injury. In contrast, at two weeks post-injury, there was a large increase in the number of 3H-T proliferating cells in the brain, with accompanying hippocampal weight increases. Both studies suggest that neonatal brain injury (e.g., hypoxia, ischemia) activates endogenous neurogenesis in the brain and promotes the repair of neurological damage, specifically in the SGZ and SVZ. However, literature (29) pointed out that the capacity for spontaneous endogenous neurogenesis is limited and insufficient for replacing neurons lost to brain damage. Therefore, it is of great clinical value and social significance to seek therapeutic techniques that promote endogenous neurogenesis, to reduce neonatal neurological dysfunction from HIBD.

## Molecular mechanisms involved in endogenous neurogenesis after neonatal HIBD

### HIF-1

Hypoxia-inducible factor (HIF-1) is a major transcriptional activator induced by hypoxia and ischemia. In hypoxic conditions, HIF-1 acts as the main mediator of a series of *in vivo* pathophysiological responses, including angiogenesis, cell proliferation, and transcriptional induction of survival genes (30). The main function of the HIF-1 nucleoprotein is to coordinate the organism's homeostatic balance of developmental and pathological oxygen states; it does so as a heterodimer with α and β subunits. HIF-1β is stably expressed intracellularly, whereas HIF-1α contains a transcription-binding domain and is regulated by hypoxic signals. α subunits determine the biological activity of HIF-1β, whereas cellular oxygen concentration strictly regulates the expression of HIF-1α (31). Some studies (32–34) have shown that HIF-1 gene expression is enhanced in neonatal rats after the onset of hypoxic-ischemic encephalopathy, mainly manifesting as enhanced HIF-1 expression at both the mRNA and protein levels in brain tissue. Further, immunohistochemical results suggest that enhanced expression is more pronounced in vascular endothelial cells. These cumulative findings suggest that HIF-1 may be a primary mediator following neonatal HIBD.

Erythropoietin (EPO) is the first protein that has been identified downstream of HIF-1 to activate erythropoiesis (35). In hypoxic conditions, elevated EPO expression promotes erythropoiesis, enhancing oxygen transport and thus adaptation to the hypoxic environment (36). Though it was previously thought that EPO was solely a key gene in the maturation and proliferation of red lineage progenitors, later studies showed that it is widely expressed in mammalian brain cells, including neurons and glial cells, which also express EPO receptors (37). Additionally, HIF-1 activates increased EPO expression, to induce expression of brain-derived neurotrophic factor (BDNF) and promote hippocampal neurogenesis (27). This cumulative evidence indicates that following injury from neonatal HIBD, the brain activates HIF-1-mediated factors, which promote endogenous neurogenesis.

### Shh signaling pathway

The sonic hedgehog (Shh) signaling pathway plays an important role in endogenous neurogenesis (38) and can play a neuroprotective role in ischemia-exposed brain injury through the activation of pathway proteins (39). This pathway regulates NSCs growth, survival, and differentiation by upregulating the expression of the transcription factor Gli1 in the SVZ. Gli1, which exerts a neuroprotective effect, induces the production of ganglionic neurons in the medial and lateral forebrain. In the midbrain and hindbrain, it induces the production of 5-hydroxytryptaminergic and dopaminergic neurons (40). It also plays an important role in NSCs renewal and migration (41). During normal development, Shh signaling pathway activation can establish homologous domains in the dorsal-ventral axis, to organize neural tube development across regions and maintain brain functional integrity (42). After brain injury, the Shh signaling pathway induces SVZ progenitor cells to produce large amounts of transcription factors, which determine cell differentiation types through time-dependent mechanisms and concentration gradients, compensating for brain cell losses (43). Thus, Shh signaling pathway expression upregulation may be a primary mechanism of endogenous neurogenesis following neonatal HIBD.

### Notch pathway

The Notch signaling pathway is a highly conserved signaling pathway that plays a critical role in the process of endogenous neurogenesis (44). Under normal conditions, the Notch signaling pathway is relatively inhibited and is activated when ischemic changes occur, playing an active role in immune inflammation, neurogenesis, and apoptosis (45). A study (46) showed that after neonatal HIBD, the expression levels of Notch1 and its downstream signaling molecule Hes1 were found to be upregulated and the number of newborn neurons in the SGZ region of the brain increased; while the number of newborn neurons in this region decreased after the application of Notch signaling pathway inhibitors, suggesting that the activation of the Notch signaling pathway can promote the process of endogenous neurogenesis induced by HIBD.

### PI3K pathway

The Phosphatidylinositol-3-kinase (PI3K) pathway has already been identified to play an important role in cell survival of endogenous neurogenesis and many studies have focused on use of the PI3K pathway to treat brain injury after stroke (47). A recent study (48) has shown that PI3K activating its downstream effector protein kinase B (Akt) can improve hippocampal brain injuries and restore neuronal development after neonatal HIBD, which demonstrates that the activation of PI3K/Akt pathway can promote endogenous neurogenesis induced by HIBD.

### Wnt/β-catenin pathway

Wingless-type mouse mammary tumor virus integration site/β-catenin (Wnt/β-catenin) signaling is a vital pathway for endogenous neurogenesis and an essential signaling system during embryonic development and aging (49). Most studies demonstrate that Wnt/β-catenin regulates progenitor self-renewal but others suggest it can also promote differentiation (50). A recent study (51) has shown that some treatments could alleviate the neurological deficits after neonatal HIBD by up-regulation of β-catenin protein in the brain. Taken together, the Wnt/β-catenin pathway also plays a crucial role in the endogenous neurogenesis after neonatal HIBD.

### Other influencing factors

The process of neurogenesis after HIBD is made up of multiple signaling molecules and pathways that are interconnected, coordinated, and work together to regulate the continuous process of neurogenesis. Several other modulators have been found to promote neurogenesis, reduce the size of infarcts and promote neurological recovery in the brain after HIBD, as follows: Vascular endothelial growth factor (VEGF) binds to the VEGFR2 receptor and activates the Rho/Rok pathway, promoting synaptic growth and playing a crucial role in neuroprotection and neurogenesis (52, 53). BDNF activates glutamatergic neurons and subsequently promotes endogenous neurogenesis after HIBD *via* the BDNF-TrkB-CREB signaling pathway (54).

## Therapeutic measures for neonatal HIBD to promote endogenous neurogenesis

The ability of spontaneous endogenous neurogenesis after neonatal HIBD to repair damaged neurological functions is markedly limited. Therefore, developing therapeutic techniques (Table 1) to promote endogenous neurogenesis to repair neurological deficits after neonatal HIBD is an urgent priority.

**Table 1:** Therapeutic approaches to promote endogenous neurogenesis and its mechanisms. HIBD, Hypoxic-ischemic brain damage; NSCs, Neural stem cells; EE, Enriched environment; PFT-α, Pifithrin-α; BDNF, Brain-derived neurotrophic factor; VEGF, Vascular endothelial growth factor.

Therapeutic measures to promote endogenous neurogenesis		Mechanisms associated with neurogenesis	Study
Therapeutic hypothermia		Short-term, has a positive effect on endogenous neurogenesis after neonatal HIBD by inhibiting the classical complement pathway, weakening neuroinflammation and thus promoting neurogenesis	(55–57)
Neural stem cells therapy		NSCs transplantation significantly reduces lesion volume in the acute phase and preventes neuron loss in the chronic phase afeter HIBD	(58, 59)
Exosomes therapy		Astrocyte-derived exosomes could carry miR-17-5p to protect neonatal rats from HIBD *via* regulating endogenous neurogenesis.	(60)
Hyperbaric oxygen therapy		Promotes NSCs proliferation through the VEGF/ERK signaling pathway	(61)
EE intervention		An intervention that promotes neurogenesis and functional recovery after ischemic brain injury and contributes to neuroprotection by upregulating expression of HIF-1α in brain tissue	(62, 63)
**Medications**	**Drug name**		
Anti-apoptotic drugs	PFT-α and Z-DEVD-fmk	Improve survival of endogenous NSCs after HIBD by inhibiting apoptosis-related signaling pathways, thereby promoting the process of endogenous neurogenesis	(64, 65)
	Atorvastatin	Promotes proliferation, differentiation and survival of NSCs by activating the PI3K/AKT and ERK pathways	(66)
Anti-inflammatory drugs	Minocycline	Alleviates depression-like symptoms by rescuing decrease in neurogenesis in dorsal hippocampus *via* blocking microglia activation	(67)
Endogenous growth factor analogues	BDNF and its analogues GSB-106 and GSB-214	Upregulate the PI3K/Akt and ERK pathways to promote endogenous neurogenesis after brain injury and improve neurological function	(68, 69)
	Epidermal growth factor, fibroblast growth factor	Promote proliferation and survival of NSCs after HIBD, promoting endogenous neurogenesis	(70)

### Therapeutic hypothermia

Several clinical studies have shown that hypothermia initiated at less than 6 h after birth reduces death or disability for infants with HIBD at 36 weeks or later gestation (8) and hypothermia treatment could improve patients' neurodevelopment two years after HIBD (7); however, whether therapeutic hypothermia facilitates neonatal endogenous neurogenesis after HIBD remains uncertain. One study (55) indicated that prolonged (>24 h) sub-hypothermia reduces cell proliferation in the SGZ, but not the SVZ, in neonatal rats. In contrast, a newer study (56) showed that a short period (4 h) of therapeutic sub-hypothermia partially rescued SGZ NSCs from apoptosis and increased the proliferation of SGZ neural precursor cells. Therefore, care should be taken in the selection of appropriate temperature and time parameters for therapeutic hypothermia in neonates following HIBD, as short periods may have a positive effect on endogenous neurogenesis, while longer periods may have the opposite effect. Currently, the clearest neuroprotective mechanism of action of therapeutic hypothermia is that it inhibits the classical complement pathway following HIBD, thereby reducing neurological damage from neuroinflammation (57).

### Neural stem cells therapy

Stem cell-based treatments for HIBD have shown promising therapeutic efficacy in preclinical studies (17). Endogenous NSCs have the self-repair ability after brain injury but it is usually insufficient and needs time to proliferate and migrate to the lesion area. Therefore, transplantation of exogenous NSCs is probably a more efficient way to improve brain restoration after injury. Some studies point out that NSCs transplantation significantly reduces lesion volume in the acute phase and prevents neuron loss in the chronic phase after HIBD (58, 59). Therefore, NSCs transplantation therapy may be one of the most promising therapeutic measures for neurologic deficits induced by HIBD.

### Exosomes therapy

Exosomes are endosomal origin membrane-enclosed small vesicles that contain various molecular constituents including proteins, lipids, mRNAs and microRNAs (71). Many studies have shown that exosomes play a crucial role in neurogenesis and are even of potential significance in treating some neurological diseases (72). A recent study (60) has pointed out that astrocyte-derived exosomes could carry miR-17-5p to protect neonatal rats from HIBD *via* regulating endogenous neurogenesis.

### Hyperbaric oxygen therapy

Hyperbaric oxygen therapy, reported to have neuroprotective effects in multiple neurological disorders, is now being evaluated as a novel Adjuvant therapy to clinical management of neonatal HIBD (73). This technique restores the blood-brain barrier *via* cerebral vasoconstriction, decreasing cerebral blood flow and reducing cerebral hematoma, and simultaneously increasing oxygen supply to ischemic tissues (74). A meta-analysis study (75) pointed out that Hyperbaric oxygen therapy significantly improved the total efficiency of treatment for neonatal HIE patients and reduced the risk of sequelae, however, the underlying mechanism is not clear. One study (76) showed that the likelihood of neurological dysfunctions is lower in a HIBD neonatal rat model undergoing hyperbaric oxygen treatment, compared with controls, suggesting that this therapy may improve prognosis *via* neuroprotective effects. Thus, hyperbaric oxygen therapy may also improve prognoses in children with HIBD by promoting endogenous neurogenesis. One paper (61) reported that hyperbaric oxygen promotes NSCs proliferation *via* the VEGF/ERK signaling pathway after traumatic brain injury, implying that it might improve prognosis following HIBD by promoting endogenous neurogenesis.

### Enriched environment intervention

Enriched environment (EE) encompasses both social interaction and the surrounding environment, based on the principle of maximizing opportunities for multisensory stimulation, voluntary physical activity, and social stimulation. EE is an intensive intervention (62) that can promote neurogenesis and functional recovery after cerebral ischemic injury, and is involved in neurological protection by upregulating HIF-1α expression in brain tissue (63). Such research (77) has used a combination of EE stimulation and G-CSF in a neonatal rat HIBD model, showing that rats in an intervention group had significantly better adaptive, fine motor, and gross motor developmental indicators compared with the control group. These studies suggest that EE interventions may promote endogenous neurogenesis, leading to long-term improvements in neurological deficits among children with HIBD.

### Anti-apoptotic drugs

High expression of the pro-apoptotic protein p53 in NSCs leads to disruption of endogenous neurogenesis after neonatal HIBD (78). Accordingly, anti-apoptotic drugs can maintain endogenous NSCs survival. The p53 pathway inhibitor pifithrin-α (PFT-α) (64) and the pro-apoptotic protein caspase-3 inhibitor Z-DEVD-fmk (65) significantly improve endogenous NSCs survival after neonatal HIBD, thus promoting endogenous post-injury neurogenesis in the brain. Ample evidence (79) shows that PI3K/Akt and ERK play important roles in the process of endogenous neurogenesis as key anti-apoptotic signaling pathways, and that drugs activating these pathways promote endogenous neurogenesis by maintaining NSCs survival. For example, the anti-apoptotic drug atorvastatin (66) promotes proliferation, differentiation, and survival of NSCs by activating the PI3K/AKT and ERK pathways. Accordingly, drugs that act on anti-apoptotic pathways are expected to be clinically targeted for the treatment of neurological dysfunction after neonatal HIBD.

### Anti-inflammatory drugs

Neuroinflammation has been demonstrated to inhibit neurogenesis and the presence of various inflammatory components, such as immune cells, cytokines, or chemokines, plays a role in regulating the survival, proliferation, and maturation of NSCs (80). It has been reported some anti-inflammatory drugs like minocycline (67) could alleviate depression-like symptoms by rescuing a decrease in neurogenesis in the dorsal hippocampus *via* blocking microglia activation, which may also apply to neonatal HIBD, because neuroinflammation is also one of the important injuries mechanisms of neonatal HIBD (81).

### Endogenous growth factor analogues

Numerous growth factors can increase the proliferation of endogenous NSCs under a variety of conditions, thus promoting the endogenous neurogenesis process. For example, BDNF and its analogues GSB-106 and GSB-214 can upregulate the PI3K/Akt and ERK pathways to promote endogenous neurogenesis after brain injury and improve neurological function (68, 69). Other growth factors like epidermal growth factor, fibroblast growth factor, and VEGF can also promote NSCs proliferation and survival post-HIBD, thus promoting endogenous neurogenesis (70).

## Conclusion

Currently, HIBD remains the leading cause of neonatal death and long-term neurological dysfunction. There is no effective treatment for the neurological sequelae caused by HIBD, neurogenesis is a promising therapeutic target for preventing HIBD-induced neurological sequelae in neonates. All of the treatments discussed above may improve the prognosis of neonatal HIBD through the target of neurogenesis. Thus, the search for optimized combined neuroprotective treatments is urgent. Moving forward, the physiological processes of endogenous neurogenesis, their molecular pathways, and the intervention mechanisms of novel technologies should be investigated in greater depth. In these ways, the most appropriate treatments and technologies can be identified to alleviate the sequelae of neurological damage in children with HIBD, with a goal of also alleviating a heavy burden on their families and society.

## References

[1] Massaro AN Wu YW Bammler TK Comstock B Mathur A McKinstry RC Plasma biomarkers of brain injury in neonatal hypoxic-ischemic encephalopathy. J Pediatr. (2018) 194:67–75.e1. 10.1016/j.jpeds.2017.10.060 29478510

[2] Pazandak C McPherson C Abubakar M Zanelli S Fairchild K Vesoulis Z. Blood pressure profiles in infants with hypoxic ischemic encephalopathy (Hie), response to dopamine, and association with brain injury. Front Pediatr. (2020) 8:512. 10.3389/fped.2020.00512 32984221 PMC7479124

[3] Vega-Del-Val C Arnaez J Caserío S Gutiérrez EP Benito M Castañón L Temporal trends in the severity and mortality of neonatal hypoxic-ischemic encephalopathy in the era of hypothermia. Neonatology. (2021) 118(6):685–92. 10.1159/000518654 34535601

[4] Novak CM Ozen M Burd I. Perinatal brain injury: mechanisms, prevention, and outcomes. Clin Perinatol. (2018) 45(2):357–75. 10.1016/j.clp.2018.01.015 29747893

[5] Peeples ES Rao R Dizon MLV Johnson YR Joe P Flibotte J Predictive models of neurodevelopmental outcomes after neonatal hypoxic-ischemic encephalopathy. Pediatrics. (2021) 147(2). 10.1542/peds.2020-022962.enc. [Epub ahead of print] 33452064

[6] Disdier C Stonestreet BS. Hypoxic-Ischemic-Related cerebrovascular changes and potential therapeutic strategies in the neonatal brain. J Neurosci Res. (2020) 98(7):1468–84. 10.1002/jnr.24590 32060970 PMC7242133

[7] Finder M Boylan GB Twomey D Ahearne C Murray DM Hallberg B. Two-year neurodevelopmental outcomes after mild hypoxic ischemic encephalopathy in the era of therapeutic hypothermia. JAMA Pediatr. (2020) 174(1):48–55. 10.1001/jamapediatrics.2019.4011 31710357 PMC6865301

[8] Laptook AR Shankaran S Tyson JE Munoz B Bell EF Goldberg RN Effect of therapeutic hypothermia initiated after 6 hours of age on death or disability among newborns with hypoxic-ischemic encephalopathy: a randomized clinical trial. JAMA. (2017) 318(16):1550–60. 10.1001/jama.2017.14972 29067428 PMC5783566

[9] Wang Q Lv H Lu L Ren P Li L. Neonatal hypoxic-ischemic encephalopathy: emerging therapeutic strategies based on pathophysiologic phases of the injury. J Matern Fetal Neonatal Med. (2019) 32(21):3685–92. 10.1080/14767058.2018.1468881 29681183

[10] Beltempo M Wintermark P Mohammad K Jabbour E Afifi J Shivananda S Variations in practices and outcomes of neonates with hypoxic ischemic encephalopathy treated with therapeutic hypothermia across tertiary nicus in Canada. J Perinatol. (2022) 42:898–906. 10.1038/s41372-022-01412-7 35552529

[11] Leiter O Zhuo Z Rust R Wasielewska JM Grönnert L Kowal S Selenium mediates exercise-induced adult neurogenesis and reverses learning deficits induced by hippocampal injury and aging. Cell Metab. (2022) 34(3):408–23.e8. 10.1016/j.cmet.2022.01.005 35120590

[12] Zalewska T Jaworska J Sypecka J Ziemka-Nalecz M. Impact of a histone deacetylase inhibitor-trichostatin a on neurogenesis after hypoxia-ischemia in immature rats. Int J Mol Sci. (2020) 21(11):3808. 10.3390/ijms21113808 PMC7312253 32471267

[13] Niklison-Chirou MV Agostini M Amelio I Melino G. Regulation of adult neurogenesis in mammalian brain. Int J Mol Sci. (2020) 21(14):4869. 10.3390/ijms21144869 PMC7402357 32660154

[14] Fares J Bou Diab Z Nabha S Fares Y. Neurogenesis in the adult hippocampus: history, regulation, and prospective roles. Int J Neurosci. (2019) 129(6):598–611. 10.1080/00207454.2018.1545771 30433866

[15] Liao LY Lau BW Sánchez-Vidaña DI Gao Q. Exogenous neural stem cell transplantation for cerebral ischemia. Neural Regen Res. (2019) 14(7):1129–37. 10.4103/1673-5374.251188 30804235 PMC6425845

[16] Gonçalves JT Schafer ST Gage FH. Adult neurogenesis in the hippocampus: from stem cells to behavior. Cell. (2016) 167(4):897–914. 10.1016/j.cell.2016.10.021 27814520

[17] Huang L Zhang L. Neural stem cell therapies and hypoxic-ischemic brain injury. Prog Neurobiol. (2019) 173:1–17. 10.1016/j.pneurobio.2018.05.004 29758244 PMC6249121

[18] Namchaiw P Wen H Mayrhofer F Chechneva O Biswas S Deng W. Temporal and partial inhibition of Gli1 in neural stem cells (Nscs) results in the early maturation of Nsc derived oligodendrocytes in vitro. Stem Cell Res Ther. (2019) 10(1):272. 10.1186/s13287-019-1374-y 31455382 PMC6712625

[19] Leuner B Sabihi S. The birth of new neurons in the maternal brain: hormonal regulation and functional implications. Front Neuroendocrinol. (2016) 41:99–113. 10.1016/j.yfrne.2016.02.004 26969795 PMC4942360

[20] Li YD Luo YJ Chen ZK Quintanilla L Cherasse Y Zhang L Hypothalamic modulation of adult hippocampal neurogenesis in mice confers activity-dependent regulation of memory and anxiety-like behavior. Nat Neurosci. (2022) 25(5):630–45. 10.1038/s41593-022-01065-x 35524139 PMC9287980

[21] Sha H Peng P Wei G Wang J Wu Y Huang H. Neuroprotective effects of dexmedetomidine on the ketamine-induced disruption of the proliferation and differentiation of developing neural stem cells in the subventricular zone. Front Pediatr. (2021) 9:649284. 10.3389/fped.2021.649284 34386466 PMC8353121

[22] Xie F Liu H Liu Y. Adult neurogenesis following ischemic stroke and implications for cell-based therapeutic approaches. World Neurosurg. (2020) 138:474–80. 10.1016/j.wneu.2020.02.010 32147554

[23] Mijajlović MD Pavlović A Brainin M Heiss WD Quinn TJ Ihle-Hansen HB Post-stroke dementia - a comprehensive review. BMC Med. (2017) 15(1):11. 10.1186/s12916-017-0779-7 28095900 PMC5241961

[24] Bielefeld P Durá I Danielewicz J Lucassen PJ Baekelandt V Abrous DN Insult-induced aberrant hippocampal neurogenesis: functional consequences and possible therapeutic strategies. Behav Brain Res. (2019) 372:112032. 10.1016/j.bbr.2019.112032 31199935

[25] Shin JE Lee H Jung K Kim M Hwang K Han J Cellular response of ventricular-subventricular neural progenitor/stem cells to neonatal hypoxic-ischemic brain injury and their enhanced neurogenesis. Yonsei Med J. (2020) 61(6):492–505. 10.3349/ymj.2020.61.6.492 32469173 PMC7256006

[26] Plane JM Liu R Wang TW Silverstein FS Parent JM. Neonatal hypoxic-ischemic injury increases forebrain subventricular zone neurogenesis in the mouse. Neurobiol Dis. (2004) 16(3):585–95. 10.1016/j.nbd.2004.04.003 15262271

[27] Herrera MI Kobiec T Kölliker-Frers R Otero-Losada M Capani F. Synaptoprotection in perinatal asphyxia: an experimental approach. Front Synaptic Neurosci. (2020) 12:35. 10.3389/fnsyn.2020.00035 33071771 PMC7539062

[28] Scheepens A Wassink G Piersma MJ Van de Berg WD Blanco CE. A delayed increase in hippocampal proliferation following global asphyxia in the neonatal rat. Brain Res Dev Brain Res. (2003) 142(1):67–76. 10.1016/S0165-3806(03)00032-4 12694945

[29] Donega V van Velthoven CT Nijboer CH Kavelaars A Heijnen CJ. The endogenous regenerative capacity of the damaged newborn brain: boosting neurogenesis with mesenchymal stem cell treatment. J Cereb Blood Flow Metab. (2013) 33(5):625–34. 10.1038/jcbfm.2013.3 23403379 PMC3652688

[30] Singh D Arora R Kaur P Singh B Mannan R Arora S. Overexpression of hypoxia-inducible factor and metabolic pathways: possible targets of cancer. Cell Biosci. (2017) 7:62. 10.1186/s13578-017-0190-2 29158891 PMC5683220

[31] Thomas LW Ashcroft M. Exploring the molecular interface between hypoxia-inducible factor signalling and mitochondria. Cell Mol Life Sci. (2019) 76(9):1759–77. 10.1007/s00018-019-03039-y 30767037 PMC6453877

[32] Carrica L Li L Newville J Kenton J Gustus K Brigman J Genetic inactivation of hypoxia inducible factor 1-alpha (Hif-1α) in adult hippocampal progenitors impairs neurogenesis and pattern discrimination learning. Neurobiol Learn Mem. (2019) 157:79–85. 10.1016/j.nlm.2018.12.002 30521851 PMC6389421

[33] Chu HX Jones NM. Changes in hypoxia-inducible factor-1 (Hif-1) and regulatory prolyl hydroxylase (Phd) enzymes following hypoxic-ischemic injury in the neonatal rat. Neurochem Res. (2016) 41(3):515–22. 10.1007/s11064-015-1641-y 26108712

[34] Li G Zhao M Cheng X Zhao T Feng Z Zhao Y Fg-4592 improves depressive-like behaviors through Hif-1-mediated neurogenesis and synapse plasticity in rats. Neurotherapeutics. (2020) 17(2):664–75. 10.1007/s13311-019-00807-3 31820273 PMC7283439

[35] Dzhalilova DS Diatroptov ME Tsvetkov IS Makarova OV Kuznetsov SL. Expression of Hif-1α, Nf-Κb, and Vegf genes in the liver and blood serum levels of Hif-1α, erythropoietin, Vegf, Tgf-*Β*, 8-isoprostane, and corticosterone in wistar rats with high and low resistance to hypoxia. Bull Exp Biol Med. (2018) 165(6):781–5. 10.1007/s10517-018-4264-x 30353332

[36] Razak A Hussain A. Erythropoietin in perinatal hypoxic-ischemic encephalopathy: a systematic review and meta-analysis. J Perinat Med. (2019) 47(4):478–89. 10.1515/jpm-2018-0360 30789826

[37] Wei S Luo C Yu S Gao J Liu C Wei Z Erythropoietin ameliorates early brain injury after subarachnoid haemorrhage by modulating microglia polarization via the Epor/Jak2-Stat3 pathway. Exp Cell Res. (2017) 361(2):342–52. 10.1016/j.yexcr.2017.11.002 29102603

[38] Patel SS Tomar S Sharma D Mahindroo N Udayabanu M. Targeting sonic hedgehog signaling in neurological disorders. Neurosci Biobehav Rev. (2017) 74(Pt A):76–97. 10.1016/j.neubiorev.2017.01.008 28088536

[39] Yin S Bai X Xin D Li T Chu X Ke H Neuroprotective effects of the sonic hedgehog signaling pathway in ischemic injury through promotion of synaptic and neuronal health. Neural Plast. (2020) 2020:8815195. 10.1155/2020/8815195 32802036 PMC7416279

[40] Vicario N Bernstock JD Spitale FM Giallongo C Giunta MAS Li Volti G Clobetasol modulates adult neural stem cell growth via canonical hedgehog pathway activation. Int J Mol Sci. (2019) 20(8):1991. 10.3390/ijms20081991 PMC6514872 31018557

[41] Daynac M Tirou L Faure H Mouthon MA Gauthier LR Hahn H Hedgehog controls quiescence and activation of neural stem cells in the adult ventricular-subventricular zone. Stem Cell Rep. (2016) 7(4):735–48. 10.1016/j.stemcr.2016.08.016 PMC5063572 27666792

[42] Placzek M Briscoe J. Sonic hedgehog in vertebrate neural tube development. Int J Dev Biol. (2018) 62(1-2-3):225–34. 10.1387/ijdb.170293jb 29616731

[43] Groves I Placzek M Fletcher AG. Of mitogens and morphogens: modelling sonic hedgehog mechanisms in vertebrate development. Philos Trans R Soc Lond Ser B, Biol Sci. (2020) 375(1809):20190660. 10.1098/rstb.2019.0660 32829689 PMC7482217

[44] Suzuki IK Gacquer D Van Heurck R Kumar D Wojno M Bilheu A Human-Specific Notch2nl genes expand cortical neurogenesis through delta/notch regulation. Cell. (2018) 173(6):1370–84.e16. 10.1016/j.cell.2018.03.067 29856955 PMC6092419

[45] Vieceli Dalla Sega F Fortini F Aquila G Campo G Vaccarezza M Rizzo P. Notch signaling regulates immune responses in atherosclerosis. Front Immunol. (2019) 10:1130. 10.3389/fimmu.2019.01130 31191522 PMC6540611

[46] Wang X Mao X Xie L Greenberg DA Jin K. Involvement of Notch1 signaling in neurogenesis in the subventricular zone of normal and ischemic rat brain in vivo. J Cereb Blood Flow Metab. (2009) 29(10):1644–54. 10.1038/jcbfm.2009.83 19536070 PMC2810260

[47] Koh SH Lo EH. The role of the Pi3k pathway in the regeneration of the damaged brain by neural stem cells after cerebral infarction. J Clin Neurol. (2015) 11(4):297–304. 10.3988/jcn.2015.11.4.297 26320845 PMC4596106

[48] Yazdani A Howidi B Shi MZ Tugarinov N Khoja Z Wintermark P. Sildenafil improves hippocampal brain injuries and restores neuronal development after neonatal hypoxia-ischemia in male rat pups. Sci Rep. (2021) 11(1):22046. 10.1038/s41598-021-01097-6 34764335 PMC8586032

[49] Marchetti B Tirolo C L'Episcopo F Caniglia S Testa N Smith JA Parkinson's disease, aging and adult neurogenesis: Wnt/Β-catenin signalling as the key to unlock the mystery of endogenous brain repair. Aging Cell. (2020) 19(3):e13101. 10.1111/acel.13101 32050297 PMC7059166

[50] Da Silva F Zhang K Pinson A Fatti E Wilsch-Bräuninger M Herbst J Mitotic wnt signalling orchestrates neurogenesis in the developing neocortex. EMBO J. (2021) 40(19):e108041. 10.15252/embj.2021108041 34431536 PMC8488556

[51] Gao L Yang L Cui H. Gsk-3β inhibitor Tws119 alleviates hypoxic-ischemic brain damage via a crosstalk with Wnt and notch signaling pathways in neonatal rats. Brain Res. (2021) 1768:147588. 10.1016/j.brainres.2021.147588 34310937

[52] Kimura K Matsumoto K Ohtake H Oka JI Fujiwara H. Endogenous acetylcholine regulates neuronal and astrocytic vascular endothelial growth factor expression levels via different acetylcholine receptor mechanisms. Neurochem Int. (2018) 118:42–51. 10.1016/j.neuint.2018.04.012 29705288

[53] Sun L. F-Box and Wd repeat domain-containing 7 (Fbxw7) mediates the hypoxia inducible factor-1α (Hif-1α)/vascular endothelial growth factor (Vegf) signaling pathway to affect hypoxic-ischemic brain damage in neonatal rats. Bioengineered. (2022) 13(1):560–72. 10.1080/21655979.2021.2011635 34951343 PMC8805906

[54] Bagheri A Habibzadeh P Razavipour SF Volmar CH Chee NT Brothers SP Hdac inhibitors induce bdnf expression and promote neurite outgrowth in human neural progenitor cells-derived neurons. Int J Mol Sci. (2019) 20(5):1109. 10.3390/ijms20051109 PMC6429164 30841499

[55] Kanagawa T Fukuda H Tsubouchi H Komoto Y Hayashi S Fukui O A decrease of cell proliferation by hypothermia in the hippocampus of the neonatal rat. Brain Res. (2006) 1111(1):36–40. 10.1016/j.brainres.2006.06.112 16904084

[56] Kwak M Lim S Kang E Furmanski O Song H Ryu YK Effects of neonatal hypoxic-ischemic injury and hypothermic neuroprotection on neural progenitor cells in the mouse hippocampus. Dev Neurosci. (2015) 37(4-5):428–39. 10.1159/000430862 26087836

[57] Shah TA Pallera HK Kaszowski CL Bass WT Lattanzio FA. Therapeutic hypothermia inhibits the classical complement pathway in a rat model of neonatal hypoxic-ischemic encephalopathy. Front Neurosci. (2021) 15:616734. 10.3389/fnins.2021.616734 33642979 PMC7907466

[58] Braccioli L Heijnen CJ Coffer PJ Nijboer CH. Delayed administration of neural stem cells after hypoxia-ischemia reduces sensorimotor deficits, cerebral lesion size, and neuroinflammation in neonatal mice. Pediatr Res. (2017) 81(1-1):127–35. 10.1038/pr.2016.172 27632779

[59] Herz J Köster C Reinboth BS Dzietko M Hansen W Sabir H Interaction between hypothermia and delayed mesenchymal stem cell therapy in neonatal hypoxic-ischemic brain injury. Brain Behav Immun. (2018) 70:118–30. 10.1016/j.bbi.2018.02.006 29454023

[60] Du L Jiang Y Sun Y. Astrocyte-derived exosomes carry microrna-17-5p to protect neonatal rats from hypoxic-ischemic brain damage via inhibiting Bnip-2 expression. Neurotoxicology. (2021) 83:28–39. 10.1016/j.neuro.2020.12.006 33309839

[61] Yang Y Wei H Zhou X Zhang F Wang C. Hyperbaric oxygen promotes neural stem cell proliferation by activating vascular endothelial growth factor/extracellular signal-regulated kinase signaling after traumatic brain injury. Neuroreport. (2017) 28(18):1232–8. 10.1097/WNR.0000000000000901 28953090

[62] Zhang Y Xu D Qi H Yuan Y Liu H Yao S Enriched environment promotes post-stroke neurogenesis through Nf-Κb-mediated secretion of Il-17a from astrocytes. Brain Res. (2018) 1687:20–31. 10.1016/j.brainres.2018.02.030 29481794

[63] Wu X Liu S Hu Z Zhu G Zheng G Wang G. Enriched housing promotes post-stroke neurogenesis through calpain 1-Stat3/Hif-1α/Vegf signaling. Brain Res Bull. (2018) 139:133–43. 10.1016/j.brainresbull.2018.02.018 29477834

[64] Turcato F Kim P Barnett A Jin Y Scerba M Casey A Sequential combined treatment of pifithrin-Α and posiphen enhances neurogenesis and functional recovery after stroke. Cell Transplant. (2018) 27(4):607–21. 10.1177/0963689718766328 29871513 PMC6041885

[65] Zhang JC Xu H Yuan Y Chen JY Zhang YJ Lin Y Delayed treatment with green tea polyphenol egcg promotes neurogenesis after ischemic stroke in adult mice. Mol Neurobiol. (2017) 54(5):3652–64. 10.1007/s12035-016-9924-0 27206430

[66] Choi NY Kim JY Hwang M Lee EH Choi H Lee KY Atorvastatin rejuvenates neural stem cells injured by oxygen-glucose deprivation and induces neuronal differentiation through activating the Pi3k/Akt and erk pathways. Mol Neurobiol. (2019) 56(4):2964–77. 10.1007/s12035-018-1267-6 30073508

[67] Bassett B Subramaniyam S Fan Y Varney S Pan H Carneiro AMD Minocycline alleviates depression-like symptoms by rescuing decrease in neurogenesis in dorsal hippocampus via blocking microglia activation/phagocytosis. Brain Behav Immun. (2021) 91:519–30. 10.1016/j.bbi.2020.11.009 33176182

[68] Gudasheva TA Povarnina P Logvinov IO Antipova TA Seredenin SB. Mimetics of brain-derived neurotrophic factor loops 1 and 4 are active in a model of ischemic stroke in rats. Drug Des Devel Ther. (2016) 10:3545–53. 10.2147/DDDT.S118768 27843294 PMC5098525

[69] Schäbitz WR Steigleder T Cooper-Kuhn CM Schwab S Sommer C Schneider A Intravenous brain-derived neurotrophic factor enhances poststroke sensorimotor recovery and stimulates neurogenesis. Stroke. (2007) 38(7):2165–72. 10.1161/STROKEAHA.106.477331 17510456

[70] Lu J Manaenko A Hu Q. Targeting adult neurogenesis for poststroke therapy. Stem Cells Int. (2017) 2017:5868632. 10.1155/2017/5868632 28808445 PMC5541797

[71] Yang Y Ye Y Su X He J Bai W He X. Mscs-derived exosomes and neuroinflammation, neurogenesis and therapy of traumatic brain injury. Front Cell Neurosci. (2017) 11:55. 10.3389/fncel.2017.00055 28293177 PMC5329010

[72] Reza-Zaldivar EE Hernández-Sapiéns MA Gutiérrez-Mercado YK Sandoval-Ávila S Gomez-Pinedo U Márquez-Aguirre AL Mesenchymal stem cell-derived exosomes promote neurogenesis and cognitive function recovery in a mouse model of Alzheimer's disease. Neural Regen Res. (2019) 14(9):1626–34. 10.4103/1673-5374.255978 31089063 PMC6557105

[73] Sankaran R Radhakrishnan K Sundaram KR. Hyperbaric oxygen therapy in patients with hypoxic ischemic encephalopathy. Neurol India. (2019) 67(3):728–31. 10.4103/0028-3886.263236 31347544

[74] Wei L Ren Q Zhang Y Wang J. Effects of hyperbaric oxygen and nerve growth factor on the long-term neural behavior of neonatal rats with hypoxic ischemic brain damage. Acta Cir Bras. (2017) 32(4):270–9. 10.1590/s0102-865020170040000002 28538801

[75] Gong XB Feng RH Dong HM Liu WH Gu YN Jiang XY Efficacy and prognosis of hyperbaric oxygen as adjuvant therapy for neonatal hypoxic-ischemic encephalopathy: a meta-analysis study. Front Pediatr. (2022) 10:707136. 10.3389/fped.2022.707136 35529335 PMC9069061

[76] Feng Z Liu J Ju R. Hyperbaric oxygen treatment promotes neural stem cell proliferation in the subventricular zone of neonatal rats with hypoxic-ischemic brain damage. Neural Regen Res. (2013) 8(13):1220–7. 10.4103/1673-5374.112859 25206416 PMC4107609

[77] Griva M Lagoudaki R Touloumi O Nousiopoulou E Karalis F Georgiou T Long-term effects of enriched environment following neonatal hypoxia-ischemia on behavior, Bdnf and synaptophysin levels in rat hippocampus: effect of combined treatment with G-Csf. Brain Res. (2017) 1667:55–67. 10.1016/j.brainres.2017.05.004 28495306

[78] Isoe Y Okuyama T Taniguchi Y Kubo T Takeuchi H. P53 mutation suppresses adult neurogenesis in medaka fish (oryzias latipes). Biochem Biophys Res Commun. (2012) 423(4):627–31. 10.1016/j.bbrc.2012.05.125 22659737

[79] Kisoh K Hayashi H Arai M Orita M Yuan B Takagi N. Possible involvement of Pi3-K/Akt-dependent Gsk-3β signaling in proliferation of neural progenitor cells after hypoxic exposure. Mol Neurobiol. (2019) 56(3):1946–56. 10.1007/s12035-018-1216-4 29981053

[80] Sung PS Lin PY Liu CH Su HC Tsai KJ. Neuroinflammation and neurogenesis in Alzheimer's disease and potential therapeutic approaches. Int J Mol Sci. (2020) 21(3). 10.3390/ijms21030701 PMC7037892 31973106

[81] Millar LJ Shi L Hoerder-Suabedissen A Molnár Z. Neonatal hypoxia ischaemia: mechanisms, models, and therapeutic challenges. Front Cell Neurosci. (2017) 11:78. 10.3389/fncel.2017.00078 28533743 PMC5420571

